# Treatment of Mesenchymal Stem Cell-Derived Extracellular Vesicles with Extract of Cultured *Lentinula edodes* Modulates Breast Cancer Stem Cells and MicroRNA Reprogramming

**DOI:** 10.3390/cimb48040368

**Published:** 2026-04-01

**Authors:** Farzaneh Sharifzad, Nawal Alsadi, Hamed Yasavoli-Sharahi, Roghayeh Shahbazi, Dylan Burger, Chantal Matar

**Affiliations:** 1 School of Nutrition Sciences, Faculty of Health Sciences, University of Ottawa, Ontario, ON K1N 6N5, Canada; 2 Department of Cellular and Molecular Medicine, Faculty of Medicine, University of Ottawa, Ontario, ON K1N 6N5, Canada; 3 Kidney Research Centre, The Ottawa Hospital Research Institute, Ottawa, ON K1H 8L6, Canada

**Keywords:** *Lentinula edodes*, extracellular vesicle, cancer stem cells, mesenchymal stem cell, microRNAs

## Abstract

Breast carcinoma represents the most frequent malignancy in women in Canada, and intrinsic or acquired drug resistance significantly increases the risk of recurrence and metastasis. Although front-line therapy is multimodal, chemoresistance remains a major hurdle in treatment and therapy, and the intake of natural compounds resulting from fermentation processes is currently considered an effective strategy to overcome this problem. This study investigated the effect of extracellular vesicles (EVs) from Mesenchymal Stromal/stem cells (MSCs) pretreated with cultured *Lentinula edodes* extract (AHCC) on reducing chemoresistance and modulating microRNAs in the MCF-7 and MCF-7/DOX cell lines. EV characterization was performed using nanoparticle tracking analysis, and microRNAs and the formation of cancer stem cells were studied. The miRNA analysis revealed that AHCC significantly downregulated oncogenic *miR-155* but upregulated the tumor-suppressive microRNAs *miR-34a*, *miR-Let7a*, and *miR-200c*. In vitro experiments showed inhibition of cancer stem cell proliferation after challenging the cells with AHCC-pretreated EVs. Conclusion: Our data demonstrated that AHCC may contribute to modulation of the tumor microenvironment, thus influencing the development of cancer stem cells.

## 1. Introduction

Breast cancer is the most commonly diagnosed cancer among women worldwide and remains a leading cause of cancer-related mortality. In North America, breast cancer represents a major public health burden, and according to the Canadian Cancer Society, it accounts for approximately 25% of new cancer cases and a significant proportion of cancer-related deaths among Canadian women. Similarly, recent epidemiological reports indicate that hundreds of thousands of women are diagnosed annually in the United States, highlighting the global and regional clinical significance of this disease [1,2]. Currently, the treatment protocol depends on the histology and stage of the cancer and consists of total tumor mass resection followed by one or a combination of the following treatment modalities: radical chemo-radiotherapy, hormone therapy, immunotherapy, and targeted therapy. While some patients are initially responsive to this regimen, the likelihood of recurrence is extremely high [3,4]. Moreover, metastasis and development of drug resistance represent major challenges in breast cancer therapy. Mounting evidence suggests that a sub-population of tumor cells with self-renewal properties is the primary culprit for drug resistance, metastasis, and recurrence in many types of cancer [5]. These cells exhibit epithelial–mesenchymal plasticity and dynamically interact with the tumor microenvironment, thereby driving therapy resistance, metastatic dissemination, and disease recurrence [6]. Breast cancer contains a subset of cells, named “breast cancer stem cells” (BCSCs), that are functionally enriched with stemness properties [7,8]. This small tumor sub-population significantly contributes to tumor reconstitution after therapy as well as resistance to therapy [9]. Furthermore, chemotherapeutic drugs used for treating breast cancer have significant side effects, with the potential to harm healthy cells, leading to a range of adverse effects [5,10].

Hence, breast cancer patients may benefit from nutritional interventions alongside conventional therapies by specifically targeting the regulation of cancer stem cell proliferation [11,12]. There has been an increasing focus on the usage of non-toxic natural compounds as chemo-preventive medicines for patients susceptible to tumor recurrence and/or with a high risk of metastasis, and the possible applications of mushroom extracts in cancer prevention have garnered heightened interest in recent years [13,14].

Research has demonstrated that an extract derived from cultured *Lentinula edodes* mycelia inhibits several forms of cancer, including breast cancer, as observed in both animal models and clinical investigations [15,16,17,18]. A growing body of evidence indicates that the compound AHCC has the potential to regulate cancer stem cell growth via an anti-inflammatory process. It was reported that AHCC is capable of increasing the tumor suppressor *miR-335*, thus avoiding the immune system evasion seen in cancer cells [19,20].

Moreover, numerous studies related to AHCC’s effects on immune cell modulation have reported its capacity to increase natural killer cell activity, ref. [21] decrease tumor formation [22], enhance T cell proliferation [23], alter cytokine release by modulating T cell activity [22,24,25], reduce inflammation, and prevent oxidation [26,27,28]. Furthermore, a significant number of clinical investigations have proven the safety, feasibility, and efficacy of this supplement in healthy volunteers [29,30] as well as patients with malignancies [31,32]. Evidence also indicates that AHCC regulates the immune response by sensitizing the intestinal epithelium, which is facilitated through recognition of non-pathogenic food-associated molecular patterns (FAMPS) by *TLR4* and *TLR2* receptors [33,34].

In addition to AHCC’s potential impacts on immune cells, emerging evidence suggests that AHCC may also play a role in reprogramming the tumor microenvironment [35]. Recent evidence highlights that epigenetic regulators, including non-coding RNAs such as microRNAs and long non-coding RNAs, play a central role in controlling epithelial–mesenchymal plasticity, cancer stemness, and therapy resistance, further supporting the relevance of targeting RNA-mediated regulatory networks in breast cancer [36]. This includes an impact on miRNAs, small non-coding RNAs that regulate genes through post-transcriptional regulation of gene expression, which are involved in self-renewal, progression, and metastasis [35,37]. They can be categorized, based on their function, into two distinct groups: oncomiRs, which promote tumor formation and progression and have been demonstrated to be differentially expressed in cancer cells and the tumor microenvironment [38], and miRsupps, which induce apoptosis and inhibit proliferation, invasion, and tumor growth [39].

We have previously shown that AHCC can target CSCs via epigenetic reprograming, alter some key microRNAs, and reduce the growth of functionally enriched stem cells and sphere formation [19]. The aim of this study was to investigate whether AHCC-treated MSC-derived EV-enriched fractions can modulate cancer stem cell behavior and microRNA expression in both sensitive (MCF-7) and chemoresistant (MCF-7/DOX) breast cancer models. The beneficial features of MSCs, which come from their regenerative abilities and capacity to differentiate into different cell lineages, make them a suitable model to better understand the interaction dynamics between MSCs, cancer stem cells, and the tumor microenvironment [40,41]. The clinical trial database shows that more than ten thousand trials have been registered using MSCs as the therapeutic agent in different clinical applications [42,43,44]. However, the application of these cells in cancer treatment remains controversial. While there is much evidence highlighting the potential roles of MSCs in cancer progression and pathogenesis, emerging data reveals that they could have some beneficial effects on cancer treatment. Regarding the tumor environment, MSCs can exert mutually exclusive effects on their surrounding cells, including cancer cells. In some cases, naïve MSCs have shown the ability to suppress tumors through the activity of *Wnt* signaling inhibitor protein Dickkopf-related protein 1 (DKK1) [45,46,47], leading to *c-Myc* and *Cyclin-D2* downregulation [48]. Moreover, MSCs have been shown to inhibit angiogenesis and promote the apoptosis of vascular endothelial cells [49]. On the other hand, reports have confirmed that MSCs exert adverse effects on tumor cells by upregulating the Akt/mTOR signaling pathway, enhancing angiogenesis, and promoting metastasis [50,51]. MSCs communicate with cancer or non-cancer cells in the tumor microenvironment by releasing EVs; these are nano-sized, membranous particles that serve as important bio-vehicles, traveling from original cells to recipient cells and consequently changing the fate and physiology of the recipient cells [52]. The unique characteristics of EVs have created a paradigm shift regarding their usage as therapeutic agents [53,54], with EVs derived from unmodified MSCs demonstrating significant potential in various experimental and clinical studies and showing promising therapeutic effects [55,56].

Of relevance for breast cancer is the bipartite relationship of MSC-derived EVs with cancer development and progression. The tumor-homing capacity of EVs makes them attractive agents for use as tumor-targeted delivery vehicles [57]. Several studies have reported the anti-proliferative effects of MSC-derived EVs against ovarian, bladder, hepatoma, and sarcoma tumor cells [58,59,60]. Furthermore, it has been reported that MSC-derived EVs transport different kinds of proteins, metabolites, and miRNAs to cancer cells and alter their behavior and fate by releasing their contents [61,62,63]. Therefore, MSC-derived EVs have been proposed as a promising alternative for MSCs to treat various kinds of cancers, including breast cancer [63,64].

The present study was conducted to evaluate the anti-proliferative effects of AHCC-treated MSC-derived EVs on MCF-7 (wild-type) and MCF-7/DOX cell lines.

Therefore, we evaluated the effect of conditioned MSC medium exposed to AHCC on cancer stem cells and the profile of differentially expressed miRNAs, including oncogenic (*miR-155*) and tumor suppressor miRNAs (*miR-34a*, *miR-Let7a*, *miR-200c*).

## 2. Materials and Methods

### 2.1. Chemicals and Reagents

AHCC^®^ (lot S26-0530-2)—a registered trademark of Amino Up Co., Ltd., (Sapporo, Japan)—is a standardized extract of cultured *Lentinula edodes* mycelia. The extract was prepared in Dulbecco’s modified Eagle’s medium (DMEM) (Gibco, Grand Island, NY, USA) for adherent cell culture and DMEM-F12 (Invitrogen, Carlsbad, CA, USA) for sphere culture, both at a 40 mg/mL concentration, and filtered through a 0.22 µm Millex-GV filter. For all cell treatment experiments, the stock solution was diluted in culture medium to obtain a final working concentration of 4 mg/mL. This concentration was selected based on previously published studies from our group demonstrating biological modulation of breast cancer stem cells without cytotoxic effects [19].

### 2.2. Cell Culture

Human MSCs (provided by Dr. Max Hincke, Faculty of Medicine, University of Ottawa, Canada) were cultured on a T75 tissue culture flask in DMEM, supplemented with 10% FBS and 1% penicillin/streptomycin (Invitrogen) in a humidified chamber with 5% carbon dioxide. The medium was refreshed every other day, and cells were observed daily. Serial passages were carried out when a confluent monolayer had formed.

MCF-7wt was purchased from the American Type Cell Collection (ATCC; Chicago, IL, USA), and MCF-7/DOX was gifted by Dr. Amado M. Parissenti. The cells were maintained in Dulbecco’s Modified Eagle’s medium (DMEM) containing fetal bovine serum (FBS) (10%, *v*/*v*) (Sigma-Aldrich, Oakville, ON, Canada) and penicillin/streptomycin (0.05 mg/mL) at 37 °C in a humidified atmosphere with 5% CO_2_ inside a CO_2_ incubator.

### 2.3. Spheroid Culture and Growth for Initial Assessment of AHCC

Adherent cells were detached using trypsin and counted using a Countess automated cell counter (Invitrogen). Single-cell suspensions were seeded in ultra-low attachment plates (Corning, Corning, NY, USA) at a density of 3 × 10^4^ cells per well in serum-free DMEM/F12 (1:1) medium supplemented with final concentrations of 2 mM L-glutamine, 1 mM sodium pyruvate, 10 ng/mL EGF, 20 ng/mL bFGF, 5 µg/mL insulin, 0.5 µg/mL hydrocortisone, and 1% penicillin/streptomycin.

For sphere formation assays using MCF-7 and MCF-7/DOX cells (ATCC, Manassas, VA, USA), freshly detached single cells were seeded under the same serum-free conditions in ultra-low attachment plates and cultured at 37 °C in a humidified incubator with 5% CO_2_.

Cells were treated with 4 mg/mL AHCC or a vehicle control. Spheres were allowed to form for 5–7 days and were counted under an inverted microscope. Only spheres larger than 50 µm in diameter were included in the analysis. All experiments were performed in three independent biological replicates.

### 2.4. Isolation, Characterization and Modification of EVs

MSC passages 3–8 were cultured to a cell confluency of up to 70% to isolate EVs. For the control group, the FBS-enriched medium was replaced with MSC basal medium supplemented with EV-free FBS, and for the experimental group, the MSCs were treated with EV-free FBS-enriched media plus 4 mg/mL AHCC. Forty-eight hours post-incubation with EV-free medium, the conditioned medium (CM) of each group was collected and stored at −80 °C to purify the EVs. Both groups’ conditioned media were collected 48 h post-incubation with EV-free FBS CM and EV-free FBS CM + AHCC, and they were stored at −80 °C. To purify the EVs, we used the exoEasy Maxi kit (Qiagen 76204). The isolated EVs were homogenized, pooled, and aliquoted for use in the experiments. Samples of EVs were used for the protein content assay and nanoparticle tracking analysis. Nanoparticle tracking analysis (NTA) was performed to assess the particle concentration of EV-enriched fractions using NanoSight. Samples were diluted in sterile PBS to reach the optimal detection range and analyzed under standard instrument settings. Due to the retrospective nature of the study and limitations of archival data, particle concentration (particles/mL) was the primary recorded parameter, while detailed size distribution profiles and modal diameter outputs were not retained. However, detected particles were within the expected size range for extracellular vesicle-enriched preparations.

The protein concentration of EVs was measured using a BCA protein assay kit (Pierce, ThermoFisher, Rockford, IL, USA).

### 2.5. Sphere Culture and Administration of Modified EVs

For EV administration in sphere culture, both cell lines were cultivated in the same conditions as mentioned above. Spheres were cultured in two groups: the first group (control) was treated with MSC-derived EVs, and the second (treatment group) was exposed to AHCC-treated MSC-EVs. Immediately after seeding single cells, both groups were incubated in a humidified incubator (5% CO_2_ at 37 °C).

### 2.6. In Vitro Assessment of the Immunostimulatory Effect of AHCC on Breast Cancer Cell Lines

Three samples of MCF-7 wild-type and MCF-7/DOX cells were grown in a 6-well plate at a concentration of 0.3 × 10^6^ cells/mL in DMEM complete medium. The cells were then exposed to 4 mg/mL of AHCC for 24 h, conditions that can maintain cell viability and exert immunomodulatory and epigenetic regulatory effects rather than inducing direct cytotoxicity, as reported in prior in vitro and in vivo studies [17,19]. The control groups for each cell line were also grown in an equal volume of medium. Cells were then harvested for microRNA profiling.

### 2.7. Quantitative RT-PCR for MicroRNA Profiling

RNAs were extracted from three samples of adherent and sphere cultures from both groups (control group and AHCC-exposed sphere group) using the Trizol protocol and the miRNeasy mini kit according to manufacturer’s protocol (Qiagen, Toronto, ON, Canada). The quality and quantity of extracted RNA were assessed by a Nano-drop spectrophotometer (Nanodrop Technologies, Wilmington, DE, USA).

Then, complementary DNA (cDNA) was synthesized from 2 ng/µL of a 1:60 dilution of the total RNA using a reverse transcription miRCURY LNA RT kit (Qiagen, Germantown, MD, USA). Quantitative RT-PCR was carried out with Syber-Green dye (Qiagen, Toronto, ON, Canada) in accordance with the RT-PCR system and standard PCR conditions in a CFX96 machine (Bio-Rad, Hercules, CA, USA). Primers for *miR-155*, *miR-34a*, *let-7a*, and *miR-200c* were obtained using the miRCURY LNA miRNA PCR assay system (Qiagen, Toronto, ON, Canada) as described in previous studies, and were used to assess changes in expression levels relative to the internal control *SNORD65* (hsa) [65,66,67,68].

### 2.8. Statistical Analysis

Statistical analyses were performed using GraphPad Prism (version 10), and data are presented as the mean ± SEM from three independent biological replicates (n = 3). Comparisons between two groups were performed using an unpaired two-tailed Student’s *t*-test. A *p*-value < 0.05 is considered statistically significant.

## 3. Results

### 3.1. Regulatory Effects of AHCC on Tumor Suppressor miRNA and Oncogenic miRNA Expression in Breast Cancer Cell Lines

**Figure 1:**
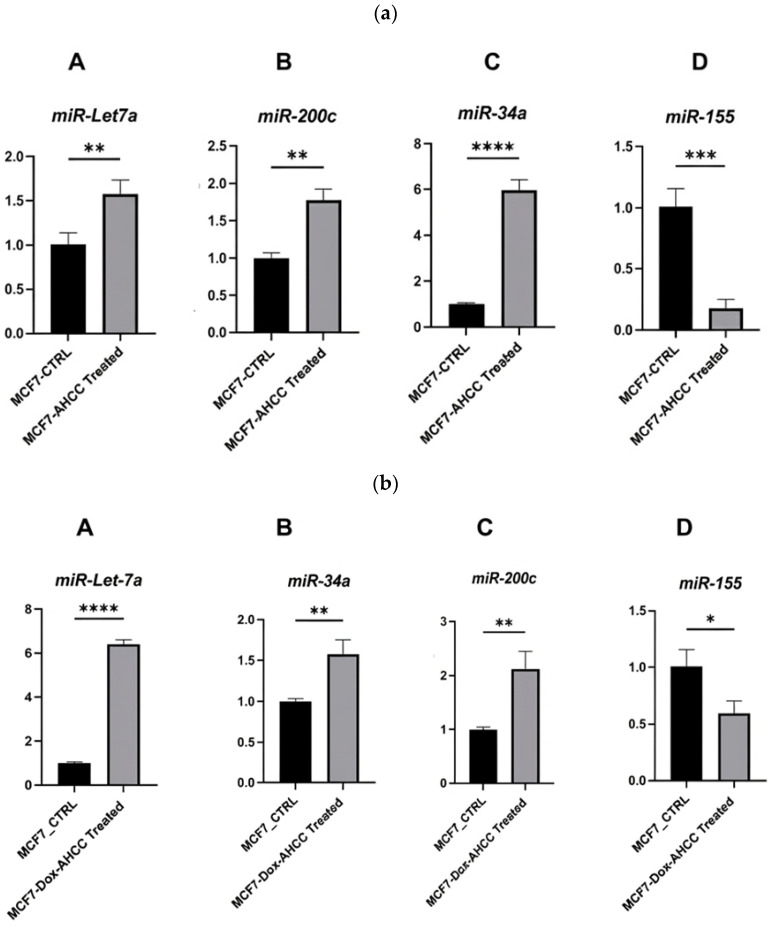
AHCC induces tumor-suppressive microRNA expression and downregulates oncogenic *miR-155* in wild-type and doxorubicin-resistant MCF-7 cells. (**a**) Quantitative RTPCR assessments of miRNA expression in wild-type MCF-7 following treatment with 4 mg/mL AHCC. Panels A, B, and C demonstrate significant upregulation of the tumor-suppressive miRNAs *miR-Let7a*, *miR-200c*, and *miR-34a*, respectively, whereas Panel D shows downregulation of oncogenic *miR-155* under the same conditions. (**b**) AHCC enhances tumor-suppressive miRNAs and downregulates *miR-155* in MCF-7/DOX cells. The graphs show the results of the comparative chronological expression analysis of miRNAs in MCF-7/DOX cell lines following treatment with a 4 mg/mL dosage of AHCC. Panel A shows higher expression levels of Let7a miRNA, Panel B shows significantly higher expression of *miR34-a* compared with the control group, Panel C depicts increased levels of *miR-200c*, and panel D shows a significant decrease in the expression of *miR-155*. Data are presented as the mean ± SEM from three independent biological replicates (n = 3), * *p* < 0.05, ** *p* < 0.01, *** *p* < 0.001, and **** *p* < 0.0001. Statistical analysis was performed using an unpaired two-tailed Student’s *t*-test.

In this investigation, we assessed the expression of miRNA in cultures obtained from wild-type MCF-7 and MCF-7/DOX cell lines. First, these cell cultures were subjected to treatment with AHCC at a dose of 4 mg/mL for 24 h. A more distinct expression pattern of microRNAs (miRNAs), namely *miR-155*, *miR-34a*, *miR-200c*, and *miR-Let7a*, was detected in the experimental group compared to the control group. It is worth mentioning that the expression of *miR-155* was dramatically reduced in the MCF-7 cells treated with AHCC in comparison to the control group, as illustrated in Figure 1a.

In contrast, there was a significant increase in the expression levels of tumor-suppressive microRNAs, including *miR-34a*, *miR-Let7a*, and *miR-200c*. The differentially expressed miRNAs in MCF-7/DOX cells treated with AHCC exhibited a similar pattern of expression (Figure 1b).

### 3.2. Isolation and Characterization of EVs

**Figure 2:**
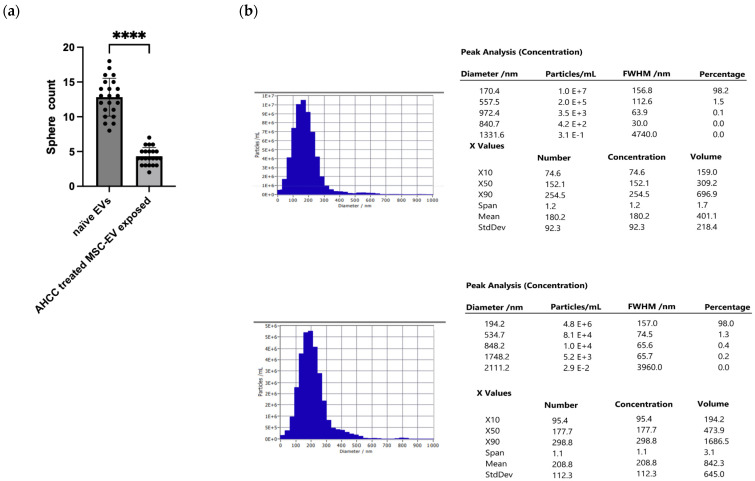
Characterization and functional effects of MSC-derived EVs. (**a**) Quantification of sphere formation in breast cancer cells treated with naïve EVs and AHCC-treated MSC-derived EVs. Data are presented as mean ± SEM (n = 3). (**b**) Nanoparticle tracking analysis (NTA) showing the particle size distribution and concentration of EVs isolated from untreated and AHCC-treated MSCs. The upper panel shows EVs from untreated MSCs (5.7 × 10^9^ particles/mL), whereas the lower panel shows EVs from AHCC-treated MSCs (1.2 × 10^10^ particles/mL). **** *p* < 0.0001.

The results of the BCA assay demonstrated a significant increase in the protein content of EVs derived from MSCs treated with AHCC. The protein concentration in the EVs from AHCC-treated MSCs was four times higher than that from untreated MSCs, with values of 0.66 µg/µL and 0.167 µg/µL, respectively. The observed increase was verified by nanoparticle tracking analysis (NTA), a technique that quantifies light scattering from EVs. NTA detected particles within the expected size range for extracellular vesicles in both preparations. The isolated EVs exhibited a mean diameter of approximately 180–210 nm, with a median size (X50) of ~150–177 nm, consistent with the typical size distribution of small extracellular vesicles (<300 nm). Furthermore, AHCC-treated MSC-derived EV fractions showed an approximately two-fold increase in particle concentration compared with untreated MSC-derived EV fractions, as presented in Figure 2 (1.2 × 10^10^ particles/mL vs. 5.7 × 10^9^ particles/mL).

### 3.3. Inhibitory Effects of AHCC-Treated MSC-Derived EVs on Sphere Formation and Sphere Growth in MCF-7 and MCF-7/Dox Cells

The impact of the MSC-derived EVs on the sphere-forming abilities of MCF-7 and MCF-7/Dox cells was also assessed. The control group consisted of EVs obtained from untreated MSCs, whereas the experimental group consisted of EVs derived from MSCs treated with AHCC. The findings demonstrated that the presence of AHCC-treated MSC-derived EVs had a suppressive effect on sphere formation and sphere growth. This observation was made during a 24-h timeframe. Subsequent inquiries were undertaken to unravel the underlying processes responsible for the observed inhibitory impact, focusing on the examination of miRNA profiles.

### 3.4. AHCC-Treated MSC-Derived EV-Enriched Fractions Were Associated with Altered Expression Levels of the Targeted MicroRNAs

**Figure 3:**
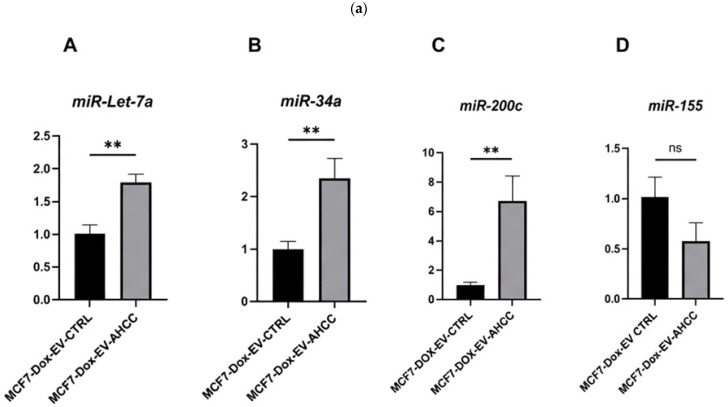

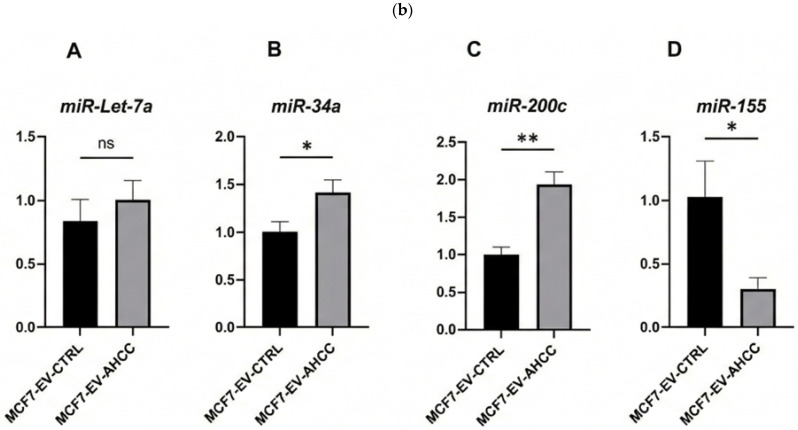
Effects of AHCC-treated MSC-derived EVs on miRNA expression in MCF-7 and MCF-7/DOX cell spheres. (**a**) AHCC-treated MSC-derived EVs upregulate tumor-suppressive miRNAs in MCF-7/DOX spheres. The graphs show the results of the quantitative RTPCR assessments of miRNA expression in MCF-7/DOX spheres following treatment with AHCC-treated and non-treated MSC-derived EVs. Panels A, B, and C demonstrate significant upregulation of the tumor-suppressive miRNAs *miR-Let7a*, *miR-34a*, and *miR-200c*, respectively. Panel D indicates that no significant difference was observed between the control group and the AHCC-treated MSC-derived EV treatment group. (**b**) Effects of AHCC-treated MSC-derived EVs on miRNA expression in MCF-7 cell spheres. The graphs show the results of the quantitative RT-PCR analysis of miRNA expression in wild-type MCF-7 cell spheres after treatment with AHCC-treated and naïve MSC-derived EVs. Panel A shows no significant difference in the expression level of Let-7a vs. the control. Panel B shows significant upregulation of *miR-34a*. Panel C shows significant upregulation of tumor-suppressive *miR-200c* expression after 24 h of exposure to AHCC-treated MSC-derived EVs. Panel D illustrates a reduction in oncogenic *miR-155* in the same sphere culture under the same conditions. Data are presented as the mean ± SEM from three independent biological replicates (n = 3), ns: significant * *p* < 0.05, ** *p* < 0.01. Statistical analysis was performed using an unpaired two-tailed Student’s *t*-test.

Using the sphere culture model, we investigated the effects of AHCC-modified MSC-derived EVs on miRNA expression in MCF-7 WT and MCF-7/Dox cell cultures. Naïve MSC-derived EVs were used as the control, and different quantities of EV proteins were utilized (5 µg/mL, 10 µg/mL, 15 µg/mL) to conduct miRNA profiling. The results revealed differential expression for all the targeted miRNAs in both cell lines when they were subjected to non-attached growing circumstances. Significantly, the administration of 10 ug/mL AHCC-modified MSC-derived EVs resulted in substantial upregulation of *miR-34a*, *miR-200c*, and *miR-let7a* expression levels within the MCF-7/Dox sphere cultures, in contrast to the MCF-7/Dox sphere cultures treated with untreated MSC-derived EVs. Although the decrease in *miR-155* expression did not reach statistical significance in the MCF-7/Dox spheres, a discernible downward trend was detected at a dose of 10 µg/mL (Figure 3a). In contrast, compared to the control group, a notable decrease in *miR-155* expression was observed at a concentration of 10 µg/mL in the MCF-7 spheres treated with extracellular vesicles derived from AHCC-modified MSCs. Moreover, notable elevations in the expression of *miR-200c* and *miR-34a* were detected in MCF-7-WT spheres (Figure 3b).

## 4. Discussion

In the continuous battle against cancer, primary, secondary, and tertiary prevention and integrative medicine have emerged as crucial components. There is a growing trend of incorporating evidence-based alternative and complementary medicine into breast cancer treatment protocols. Recently, the extract of cultured *Lentinula edodes* mycelia has garnered considerable attention as an evidence-backed component in the field of preventative medicine. Many breast cancer patients are embracing its utilization due to its potential to regulate the immune system and function as a chemo-preventive agent [66,67]. The extract is particularly enriched with the partly acylated form of α-1,4 glucan and other bioactive compounds released following culture or fermentation [26,66], and notably, we have previously shown that it can influence CSCs by modulating *miR-335* [19].

miRNAs play important roles in gene regulation in normal and disease conditions [69], and several studies have shown that dysregulated miRNA expression results in tumor development through mechanisms involved in gene expression manipulation, defects in miRNA biogenesis, and dysregulation of the cell microenvironment [70]. MicroRNAs may act as either tumor suppressors or oncogenes [71], and in this study, four miRNAs were targeted: the oncogene *miR-155*, and the tumor suppressors *miR-34a*, *miR-200c*, and *miR-Let7a*.

*miR-155* is an oncomiR known to act as an oncogene by inhibiting the anti-tumoral activity of p53 [63]. *miR-155* is significantly upregulated in breast cancer; positively correlated with high tumor grade, progressive disease, and metastases; and is inversely correlated with overall and disease-free survival. *miR-155* plays an important role in the immunopathogenesis of breast cancer, and its expression is promoted by factors that induce tumor inflammation, like interleukin IL6 and interferon IFN-γ [72]. In the present study, AHCC was able to downregulate this oncomiR in CSCs of the chemoresistance cell line, and the results showed that modified EVs can effectively decrease oncomiR-155. *miR-34a* is a tumor suppressor linked to the prevention of cancer progression [64], so its downregulation correlates with cancer progression. For instance, aberrant CpG methylation of the *miR-34a* promoter has exhibited significant reduction in several cancers [73]. A growing body of evidence has pointed to the role of *mir-34a* in cell cycle, apoptosis, and mitotic signaling [74] via the regulation of several genes involved in stemness phenotype maintenance, chemoresistance, and metastasis [73,74]. In this regard, it has been shown that oncogenic pathways (Wnt, Notch) and transcription factors such as c-Myc, Oct4, and Klf4 downregulate *miR-34a* expression in many types of cancers [36,75,76]. Moreover, computational prediction and some experimental studies propose c-Myc as a target for *miR-34a* [77]. In the current study, treating tumorspheres with AHCC led to significant *miR-34a* upregulation in MCF-7 and MCF-7/DOX spheres, as depicted in Figure 1 and Figure 2.

The let-7 family was the first family of miRNAs to be studied [78], and its downregulation has been shown to have a prognostic effect on lung cancer patients’ survival after surgery [79]. Many members of the let-7 family are considered to be tumor suppressors; however, let-7a is the most-studied miRNA, and has an impact on oncogenic c-Myc [78]. Our data indicate that AHCC upregulates let-7 expression in a dose-dependent manner and affects sphere formation in both wild-type and chemoresistant cell lines (1, 2).

*miR-200c* is a member of the miRNA family and has been shown to be a tumor suppressor in some cancers, including breast cancer [17]. It regulates epithelial–mesenchymal transition (EMT) via ZEB1/2 downregulation and E-cadherin upregulation [65], and evidence suggests that it is a novel regulator of TLR4 and HMBG-1 signaling and plays essential roles in cell cycle control, proliferation, invasion, apoptosis, and metastasis in different cancer types [80]. We assessed the expression of *miR-200c* in MCF-7 and in MCF-7/Dox treated with AHCC after 24 h, and our data confirms the effect of AHCC on *miR-200c* expression, as shown in Figure 1b and Figure 2b.

AHCC is recognized for its system modulation and preventive effects against cancer. Our study emphasizes its influence on oncomiRs and tumor-suppressing miRNAs like *miR-155*, *miR-34a*, *miR-let7a*, and *miR-200c*. It shows that AHCC reduces the expression of oncomiR *miR-155*, which hinders tumor suppressor p53 and is linked to advanced tumor stages and tumor spread. Moreover, AHCC boosts the levels of tumor-suppressing miRNAs such as *miR-34a*, *miR-let7a*, and *miR-200c*, which play roles in impeding cancer’s advancement, in epithelial–mesenchymal transition, and in inhibiting cancer growth. MCF-7 and its doxorubicin-resistant counterpart (MCF-7/DOX) were selected as a well-established paired model to investigate breast cancer chemoresistance. This model allows direct comparison between drug-sensitive and -resistant phenotypes and is widely used for studying mechanisms of therapy resistance and microRNA modulation in breast cancer.

It is important to note that the experimental design focused on comparing AHCC-treated MSC-derived EVs with naïve MSC-derived EVs as the biologically relevant control. The aim of the present study was to evaluate EV-mediated modulation of microRNA expression and cancer stem cell behavior, rather than direct cytotoxic drug activity. Therefore, a classical positive pharmacological control was not included in this experimental framework.

Despite the valuable insights provided by the findings of this study, several limitations should be acknowledged. This research used only in vitro models with MCF-7 and MCF-7/DOX cell lines to study AHCC-modified MSC-derived EVs, but their potential for translation needs to be investigated through in vivo experiments. This research found substantial changes in microRNA expression, but it did not perform direct tests to confirm which target pathways were affected by these changes. In addition, the study lacks a non-malignant mammary epithelial cell line as a control, which would help determine the specific nature of the observed effects. Future research needs to focus on these areas to better understand the relevant mechanisms and achieve improved treatment outcomes.

While significant modulation of microRNA expression was observed following exposure to AHCC-treated MSC-derived EV-enriched fractions, direct mechanistic confirmation of RNA transfer was not investigated in the present study.

In summary, this research highlights the impact of AHCC in reducing chemoresistance and modulating miRNAs in breast cancer through MSC-derived EVs.

## 5. Future Investigations

To explore how AHCC interacts with pathways related to cancer progression or inhibition, future investigations should aim to unravel the connections between AHCC and other cancer-related pathways, like p53 signaling, Wnt/β catenin, Notch, and Hedgehog.

Investigating the impact of AHCC on regulation in breast cancer cells will require studying how AHCC influences DNA methylation/demethylation patterns and histone modifications in transcription factors. Additionally, exploring how AHCC affects the characteristics of CSCs will require examining its influence on CSC maintenance, differentiation, and self-renewal abilities in relation to related transcription factors. For in vivo modeling of AHCC treatment, researchers must employ animal models to assess the therapeutic efficacy of AHCC on tumor growth and metastasis prevention. Finally, future studies should evaluate the potential synergic effects of checkpoint inhibitors like PD1/PDL1 and CTLA4 to achieve the best immunotherapeutic results.

## Data Availability

The raw data supporting the conclusions of this article will be made available by the authors on request.

## Abbreviations

The following abbreviations are used in this manuscript:

EVs	Extracellular vesicles
MSCs	Mesenchymal Stromal/stem cells
BCSCs	Breast cancer stem cells
FAMPS	Food-associated molecular patterns
